# Parasitism, life history traits and immune defence in cyprinid fish from Central Europe

**DOI:** 10.1186/1471-2148-8-29

**Published:** 2008-01-28

**Authors:** Andrea Šimková, Thomas Lafond, Markéta Ondračková, Pavel Jurajda, Eva Ottová, Serge Morand

**Affiliations:** 1 Institute of Botany and Zoology, Faculty of Science, Masaryk University, Kotlářská 2, 61137 Brno, Czech Republic; 2 Institut des Sciences de l'Evolution CNRS, CC065, Université Montpellier 2, 34095 Montpellier cedex 05 France; 3 Laboratoire de Parasitologie Evolutive, UMR 7103, Université Pierre et Marie Curie, 7, Quai St. Bernard, 75252 Paris Cedex 05, France; 4 Institute of Vertebrate Biology, Academy of Sciences of the Czech Republic, Květná 8, 60365 Brno, Czech Republic

## Abstract

**Background:**

The main prediction of life-history theory is that optimal energy allocated among the traits is related to the growth, maintenance and survival. It is hypothesized that the optimal resource allocated to immune function, which generates resistance towards parasites and reduce the fitness losses caused by parasitism, is depending on other requirements for energetic resource and the benefits associated with them. The aims of this study are to investigate in a comparative way (1) how parasitism is related to fish life history traits (fecundity, longevity, mortality), (2) whether there is a trade-off between reproduction and immune investments in fish females (i.e. energetic hypothesis) and in males (i.e. immunohandicap hypothesis), (3) whether parasitism influences host immunity (spleen size) and reproduction (gonad size) in females and males.

**Results:**

Data on metazoan parasites of 23 cyprinid fish species from Central Europe were used for the analyses as well as new data collected from a field study. Ectoparasite species richness was negatively correlated with the fish mortality estimated by the k-value and positively correlated with fish body size, suggesting that parasite diversity increases with fish longevity. A negative relationship between spleen size and gonad size, controlling for fish body size, was found in females but not in males. Moreover, parasite abundance was positively correlated with fish spleen size and negatively with fish gonad size in females.

**Conclusion:**

The comparative analyses using cyprinid fish species demonstrated that natural mortality could be considered as a factor contributing to the variability of parasite species richness and moreover, parasite species benefit from long-lived fish. The results obtained from the analyses investigating the potential trade-off between reproduction and immunity could be interpreted as an energetic trade-off between female reproduction and immune function. The lack of negative relationship between gonad size and spleen size in males did not support our prediction based on the immunohandicap hypothesis.

## Background

Parasites negatively influence host fitness, and subsequently hosts develop their anti-parasite defence, i.e. a performing immune system, in order to reduce fitness cost induced by parasitism [1]. However, an increased investment in immune defence should give rise to a trade-off with life-history traits such as growth, survival and reproduction [2]. Several studies supporting this prediction have been performed, predominantly in birds [3-5]. Fewer studies have been conducted in fish [6,7], which differ from birds and mammals in several aspects of their life history traits [8,9].

Investment in immune defence is commonly measured by spleen size, density of blood cells or as T-cell mediated immune response [3,4,7,10-14]. Several comparative studies use the relative spleen size as a potential measure of immune investment in birds against parasites [4,11,15,16], in which it was predicted that host species encountering more parasites possess larger spleens because they have more invested in immune defence [16]. Moreover, the use of spleen size is also recommended in ecotoxicological studies as a standard measure of immunocompetence [17]. Spleen plays a highly important role in hemopoiesis and immune reactivity of teleost fish producing antibodies and participating in clearance of pathogens and foreign particles from the blood stream [18]. Spleen size is widely applied as an estimator of immunocompetence in recent published studies on fish and used as a measure of investment in immunity in intra-specific analyses [7,19-22].

The impacts of parasites on host survival, reproduction and mating success are well documented in fish [23-28]. Fish males and females invest differently in reproduction with females investing more in gamete production, and males investing more in mate attraction through the display of sexual ornamentation, which are exacerbated during the spawning period [6,7].

Two hypotheses were formulated for explaining the differential investment in reproduction and immune response in link with parasite pressure. The first hypothesis postulates the existence of an energetic trade-off between the investment in reproduction and the investment in immune responses [1], with the assumption that immune response is costly and reduces the energy for other tasks such as reproduction. If this energetic hypothesis is correct, a trade-off between gonad development and spleen size in females should be observed.

The second one, the immunohandicap hypothesis [6,29-32] emphasizes the potential role of immunosuppression induced by steroid sexual hormones (mainly testosterone). These hormones and some of their precursors directly affect the production of immune cells in fish [33,34]. The increasing level of steroid hormones at the very beginning of reproduction stimulates the expression of sexual ornamentation, but negatively influences the immune function ability (i.e. immunosuppression). As a consequence, a negative effect of testosterone on the relative spleen weight is observed in fish [14]. The immunosuppression by steroid hormones could result in higher parasites intensities in breeding individuals or in individuals with high expression of sexual ornamentation [20,29]. As a consequence, a trade-off between gonad development and spleen size in males was predicted and observed [4]. From this point of view, the immunohandicap hypothesis could be also seen as a part of larger concept of energetic trade-offs between investment in reproduction and immune defence in males. In addition, a relationship between parasites and well-developed ornamentation was predicted [35,36]. This has been observed or partially confirmed in fish males [6,32,37].

The aims of this study are to investigate in a comparative way:

- how parasitism is related to fish life history traits,

- whether there is a trade-off between reproduction and immune investments in fish females (i.e. energetic hypothesis) and in males (i.e. immunohandicap hypothesis),

- whether parasitism influences host immunity (spleen size) and reproduction (gonad size) in females and males.

## Results

### Fish life history traits: allometry and trade-off

K-value, a parameter of the von Bertalanffy growth function, considered as a good predictor of natural mortality was not correlated with fish body size, whilst female fecundity was positively correlated with maximal fish size (N = 19, b = 2.41, R^2 ^= 0.75, p < 0.0001). Maximal fish longevity was also positively correlated with maximal fish size (N = 19, b = 0.66, R^2 ^= 0.65, p < 0.0001). A negative relationship between female fecundity and maximal longevity was found after correcting both variables for maximal fish body size (N = 19, b = -0.25, R^2 ^= 0.51, p = 0.0004). We used residuals of fecundity and longevity obtained from these regressions for the next analyses.

### Parasitism and fish life history traits

Data from the literature that were used are given in Table 1. Life history traits (k-value, female fecundity and maximal longevity) were tested as potential determinants of parasite species richness.

**Table 1:** List of cyprinid fish species investigated with data on host sample size, life-history traits and parasite species richness (ectoparasites, endoparasites and total metazoan parasites). K-value represents a parameter of the von Bertalanffy growth function. Female fecundity represents an average number of eggs per female in one breeding season. ? – data not available (see Material and Methods for sources of data).

Fish species	Host sample size	Female fecundity	K – value	Maximal body size (in cm)	Longevity (maximal age)	Ectoparasite species richness	Endoparasite species richness	Parasite species richness
*Abramis ballerus *(Linnaeus, 1758)	3	67850	0.191	35	8	5	3	8
*Abramis bjoerkna *(Linnaeus, 1758)	25	75000	0.27	54.5	16	10	13	23
*Abramis brama *(Linnaeus, 1758)	159	174500	0.429	75	16	13	12	25
*Alburnoides bipunctatus *(Bloch, 1782)	4	8000	0.36	15	6	1	0	1
*Alburnus alburnus *(Linnaeus, 1758)	81	6750	0.31	20	6	19	15	34
*Aspius aspius *(Linnaeus, 1758)	11	316100	0.336	100	15	7	8	15
*Barbus barbus *(Linnaeus, 1758)	8	100000	0.08	100	25	7	5	12
*Carassius auratus *(Linnaeus, 1758)	14	429100	0.284	52	8	9	5	14
*Carassius carassius *(Linnaeus, 1758)	4	300000	0.358	53	6	3	2	5
*Chondrostoma nasus *(Linnaeus, 1758)	7	21400	0.23	56.5	17	5	1	6
*Cyprinus carpio *Linnaeus, 1758	15	300000	0.396	120	30	9	3	12
*Gobio albipinnatus *Lukasch, 1933	7	?	?	12	?	2	2	4
*Gobio gobio *(Linnaeus, 1758)	64	3450	0.355	14	8	9	6	15
*Leucaspius delineatus *Heckel, 1843	2	1200	0.39	10	3	2	0	2
*Leuciscus cephalus *(Linnaeus, 1758)	108	29000	0.28	78	20	25	21	46
*Leuciscus idus *(Linnaeus, 1758)	6	34100	0.11	62	15	7	4	11
*Leuciscus leuciscus *(Linnaeus, 1758)	39	9750	0.338	35	10	7	10	17
*Phoxinus phoxinus *(Linnaeus, 1758)	11	1670	0.55	14	5	3	4	7
*Pseudorasbora parva *(Temminck and Schlegel, 1864)	12	3250	?	9	3.5	1	1	2
*Rhodeus sericeus *(Pallas, 1776)	32	160	0.32	7.5	5	4	2	6
*Rutilus rutilus *(Linnaeus, 1758)	98	65000	0.21	52	10	20	9	29
*Scardinius erythrophthalmus *(Linnaeus, 1758)	13	48900	0.527	45	15	5	3	8
*Tinca tinca *(Linnaeus, 1758)	22	400000	0.71	68	9	5	6	11

First, we found that:

- Total parasite species richness was positively correlated with host sample size (b = 0.54, p < 0.0001) and fish body size (b = 0.46, p = 0.0185) using multiple regression (N = 19, R^2 ^= 0.79, p = 0.0001).

- Ectoparasite species richness was positively correlated with host sample size (b = 0.44, p < 0.0001) and fish body size (b = 0.37, p = 0.0214) using multiple regression (N = 19, R^2 ^= 0.78, p < 0.0001).

- Endoparasite species richness was positively correlated with host sample size (b = 0.53, p < 0.0001) and fish body size (b = 0.39, p = 0.0353) using multiple regression (N = 19; R^2 ^= 0.78, p < 0.0001).

Total parasite species richness, ecto- and endoparasite species richness were corrected for both host sample size and fish body size using residuals obtained from these multiple regression.

We used the residuals of parasite species richness (total, ecto- and endoparasites) of these regressions for the next analyses.

**Figure 1:**
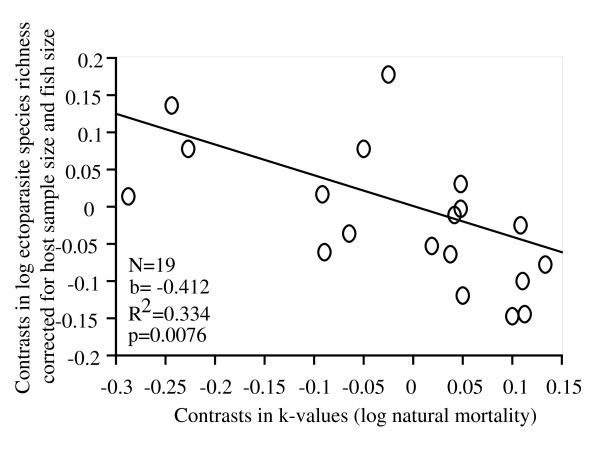
Relationship between independent contrasts of ectoparasite species richness and k-values (connected with natural mortality, see Methods). Ectoparasite species richness was controlled for host sample size and host body size (residuals from multiple regression).

Second, we tested the existence of correlation between fish life traits and parasitism. Multiple regression revealed a negative relationship between mortality estimated by k-value and residuals of ectoparasite species richness (p < 0.01, see Figure 1). This result is still significant using Bonferroni correction (p < 0.05). A negative but not significant relationship was also found between mortality and residuals of total parasite species richness (p = 0.073). No relationship was found between life-history traits and residuals of endoparasite species richness (p > 0.05).

### Parasitism, immunity and reproduction: intra-specific analysis

We performed a variance component analysis on the spleen weight (in log) of males of 4 fish species (*Barbus barbus, Leuciscus cephalus, Pseudorasbora parva, Rutilus rutilus*) and found that 82% of the variance is due to fish species. The variance component analysis of the gonad weight (in log) on males of 4 fish species showed that only 71% of the variance is due to fish species.

The ANOVA performed on males of 4 fish species (*Barbus barbus, Leuciscus cephalus, Pseudorasbora parva, Rutilus rutilus*) showed a significant effect of parasite abundance, fish weight and fish species on the gonad weight (Table 2). A marginal effect of individual parasite species richness on the gonad weight was also observed (Table 3). No effects of parasite abundance or parasite species richness were detected on spleen weight in males (all p > 0.1).

**Table 2:** Results of general linear model of the effect of fish species (four species), body weight (in log) and parasite abundance (in log) on gonad weight (in log). The ANOVA for gonad weight (in log) is highly significant (F-ratio df (5, 31) = 101.1, p < 0.0001).

Source	Sum of Squares	Df	Mean Square	F-Ratio	P-Value
Fish species	2.00	3	0.669	14.67	<0.0001
Body weight	3.60	1	3.604	78.99	<0.0001
Parasite abundance	0.35	1	0.347	7.60	0.0097
Residual	1.41	31	0.046		
Total (corrected)	24.48	36			

**Table 3:** Results of general linear model of the effect of fish species (four species), body weight (in log) and average parasite species richness (in log) on gonad weight (in log). The ANOVA for gonad weight (in log) is highly significant (F-ratio df (5, 31) = 90.6, p < 0.0001).

Source	Sum of Squares	Df	Mean Square	F-Ratio	P-Value
Fish species	2.43	3	0.809	16.00	<0.0001
Body weight	3.74	1	3.740	74.00	<0.0001
Parasite species richness	0.19	1	0.194	3.84	0.059
Residual	1.57	31	0.051		
Total (corrected)	24.48	36			

### Parasitism, reproduction and immunity: inter-specific analyses

Data from the field study are shown in Table 4. Kruskal-Wallis (KW) test revealed significant inter-specific differences for gonad weight, spleen weight, parasite abundance and parasite species richness for both males and females using raw data (for all variables p < 0.01).

**Table 4:** List of fish species with fish individuals investigated with the host sample size (females/males), total body weight, spleen weight, gonad weight, parasite abundance (mean and standard deviation are shown) and average parasite species richness. F – females, M – males.

Fish species	Host sample size	Total body	Weight (g)	Gonad	Weight (g)	Spleen	Weight (g)	Parasite	Abundance	Parasite richness	Species
		F	M	F	M	F	M	F	M	F	M
*Abramis bjoerkna*	3/6	79.1 ± 42.51	50.15 ± 32.64	11.969 ± 7.537	3.789 ± 3.779	0.14 ± 0.098	0.124 ± 0.085	114.33 ± 87.21	36.67 ± 31.69	6.33	5.17
*Abramis brama*	1/1	100.4	99.7	1.323	3.575	0.861	0.105	852	1657	1.5	3
*Alburnoides bipunctatus*	3/5	11.7 ± 4.45	10.04 ± 2.41	1.374 ± 0.517	0.900 ± 0.243	0.007 ± 0.006	0.027 ± 0.027	20 ± 18.36	13.4 ± 10.90	2.33	2.4
*Alburnus alburnus*	2/5	31.4 ± 12.45	14.8 ± 4.39	4.273 ± 2.979	1.048 ± 0.688	0.046	0.023 ± 0.010	15.5 ± 4.95	7.2 ± 4.60	5.5	3.8
*Aspius aspius*	1/0	10.8	-	0.064	-	0.022	-	29	-	2	-
*Barbus barbus*	0/9	-	52.9 ± 20.06	-	4.086 ± 1.944	-	0.133 ± 0.051	-	40.33 ± 47.41	-	3.56
*Carassius auratus*	2/3	61.45 ± 5.16	20.7 ± 3.76	8.091 ± 1.965	0.092 ± 0.076	0.143 ± 0.018	0.066 ± 0.014	54 ± 57.98	24 ± 23.06	4	3.33
*Carassius carassius*	2/2	45.15 ± 49.71	9.05 ± 8.70	3.522 ± 4.405	0.392	0.030 ± 0.023	0.027 ± 0.012	42 ± 55.15	1	2	1
*Chondrostoma nasus*	0/1	-	80.1	-	0.344	-	0.093	-	2	-	2
*Cyprinus carpio*	0/3	-	89.27 ± 14.20	-	0.148 ± 0.059	-	0.198 ± 0.072	-	15.33 ± 21.92	-	3.33
*Gobio albipinnatus*	4/1	4.58 ± 0.33	5.6	0.489 ± 0.103	0.112	0.006 ± 0.002	0.005	9.5 ± 5.47	6	1.5	2
*Gobio gobio*	3/6	3.07 ± 0.38	10.13 ± 11.66	0.355 ± 0.154	0.225 ± 0.311	0.005 ± 0.003	0.062 ± 0.085	28.67 ± 18.56	6.17 ± 5.88	2	1.17
*Leuciscus cephalus*	3/10	123.87 ± 18.87	67.9 ± 24.15	8.98 ± 4.207	1.908 ± 1.829	0.152 ± 0.059	0.122 ± 0.083	36.67 ± 21.39	19.4 ± 15.83	6.33	4.4
*Leuciscus idus*	0/3	-	21.4 ± 13.34	-	0.385	-	0.072 ± 0.023	-	3 ± 3.61	-	3.67
*Leuciscus leuciscus*	2/2	92.65 ± 11.38	77.7 ± 14.14	2.797 ± 0.052	0.878 ± 0.248	0.101 ± 0.030	0.129 ± 0.021	20.5 ± 2.12	6 ± 2.83	2	3
*Phoxinus phoxinus*	8/1	6.16 ± 3.25	3.1	0.785 ± 0.376	0.09	0.006 ± 0.004	0.009	11.5 ± 18.09	2	1.5	2
*Pseudorasbora parva*	5/8	1.2 ± 0.41	2.74 ± 1.78	0.180 ± 0.080	0.061 ± 0.047	0.002 ± 0.001	0.004 ± 0.003	0.4 ± 0.89	4.88 ± 6.38	0.2	1.25
*Rhodeus sericeus*	5/2	0.98 ± 0.26	1.45 ± 0.50	0.110 ± 0.032	0.056 ± 0.003	0.001 ± 0.001	0.003 ± 0.003	1.8 ± 1.92	0	1.6	0.5
*Rutilus rutilus*	1/10	27.1	20.26 ± 16.48	1.469	0.628 ± 0.918	0.041	0.072 ± 0.072	58	54.4 ± 73.66	7	5.3
*Scardinius erythrophthalmus*	3/2	18.77 ± 15.57	25 ± 1.70	1.588 ± 1.386	1.556 ± 0.130	0.021 ± 0.016	0.077 ± 0.018	17 ± 15.72	20 ± 11.31	3.33	3.5
*Tinca tinca*	1/1	23.1	64.2	0.29	0.609	0.175	0.4	70	2	2	2

Using independent contrasts, we found;

- positive relationships between spleen weight and fish body weight for both females (N = 15, b = 1.15, R^2 ^= 0.76, p < 0.0001) and males (N = 18, b = 1.00, R^2 ^= 0.75, p < 0.0001);

- positive relationships between gonad weight and fish body weight for both females (N = 15, b = 0.85, R^2 ^= 0.50, p = 0.0022) and males (N = 18, b = 0.50, R^2 ^= 0.24, p = 0.034).

**Figure 2:**
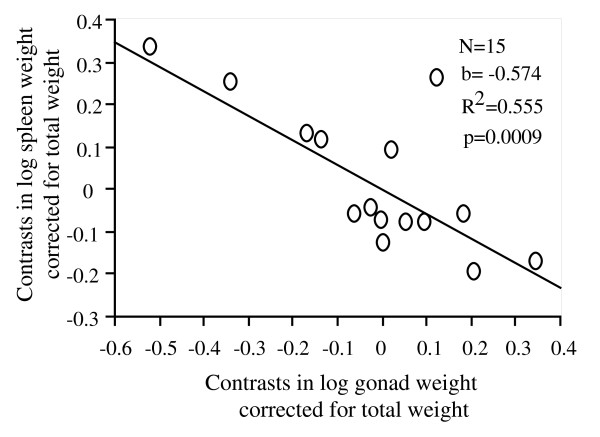
Relationship between independent contrasts of spleen weight and gonad weight in cyprinid females. Both variables were controlled for fish body weight (residuals from simple linear regression).

Residuals of spleen weight (corrected for fish body weight) was negatively correlated with residuals of gonad weight (corrected for fish body weight) in females (Figure 2, p < 0.001) but not in males (p > 0.05).

We found the same results when fish species with less than 3 individuals were removed. Moreover, the results using raw data were similar to those obtained using the independent contrasts analyses, i.e. a negative relationship was found between spleen weight and gonad weight (p < 0.05) in females but not in males (p > 0.05).

### Fish males

Parasite abundance was positively correlated with fish body weight (N = 18, b = 0.86, R^2 ^= 0.30, p = 0.0147) in males. However, no relationship was found between residuals of parasite abundance and residuals of spleen weight or residuals of gonad weight (all p > 0.05). The same results were observed when excluding fish species with less than 3 individuals. The same results were obtained using raw data (p > 0.05) excluding or not fish species with less than 3 individuals.

### Fish females

**Figure 3:**
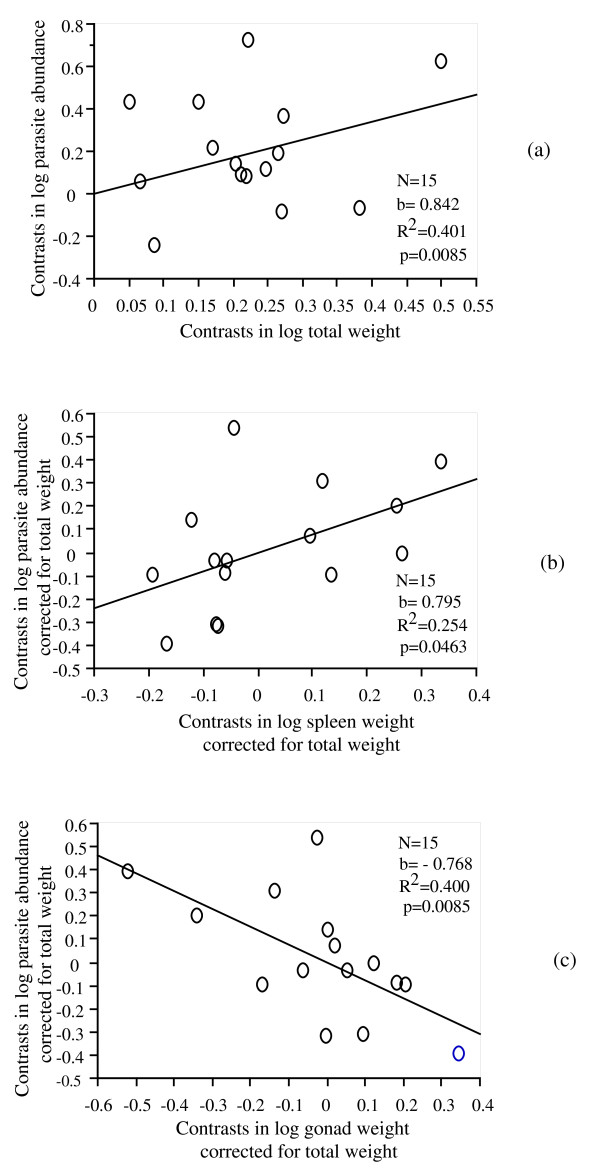
Relationship between independent contrasts of parasite abundance and female body weight (independent contrasts calculated on log-transformed values) (a). Relationship between independent contrast of parasite abundance and spleen weight in females (both variables were controlled for fish weight using residuals from linear regression) (b). Relationship between parasite abundance and gonad weight for females (both variables were controlled for fish weight using residuals from linear regression) (c). All relationships are based on data obtained from the field study on females of 17 fish species (see Methods).

Total parasite abundance was positively correlated with fish body weight (Figure 3a, p < 0.01) in females. A positive relationship was observed between residuals of total parasite abundance and residuals of spleen weight (Figure 3b, p < 0.05), whereas a negative relationship was found between residuals of total parasite abundance and residuals of gonad weight (Figure 3c, p < 0.01).

A positive significant relationship was found between residuals of ectoparasite abundance and residuals of spleen weight (N = 15, b = 1.464, R^2 ^= 0.34, p = 0.017), whereas a negative relationship was found between residuals of ectoparasite abundance and residuals of gonad weight (N = 15, b = -0.97, R^2 ^= 0.25, p = 0.047).

No relationship was observed for endoparasites (all p > 0.05) and for average parasite species richness (p > 0.05).

The comparative analyses were also performed using only fish species with at least 3 individuals, and revealed a positive trend although not significant between total parasite abundance and spleen weight (p = 0.17) and a negative trend between parasite abundance and gonad weight (p = 0.11) in females. A positive but not significant relationship between ectoparasite abundance and spleen weight (p = 0.11) and a negative relationship between ectoparasite abundance and gonad weight corrected for fish body weight was found (N = 10, b = -0.93, R^2 ^= 0.36, p = 0.0496).

Finally, the same results were obtained using raw data without correcting for phylogeny, with positive relationships between residuals of spleen weight and residuals of total parasite abundance (N = 17, b = 0.81, R^2 ^= 0.29, p = 0.025) or residuals of ectoparasite abundance (N = 17, b = 1.36, R^2 ^= 0.39, p = 0.007). These positive relationships were observed, although not significant (0.1 < p < 0.5), when excluding fish species with low sample size.

## Discussion

Evolution of life-history traits is predicted to be a consequence of how resources are allocated to competing functions such as reproduction, growth and maintenance. Therefore, the potential relationships between different fish life traits investigated in the present study, taking into account the potential influence of parasitism should be discussed in terms of trade-off. For instance high investment in reproduction might decrease energy for investment in immune functions, facilitating parasite infection [1].

### Fish life traits

In the present study we have shown several relationships among fish life-history traits previously predicted by Reznick et al. [9]. Allometric relationships with fish body size were found for maximal longevity and female fecundity. The negative relationship found between female fecundity and longevity supports the hypothesis that cyprinid fish pay for an increased investment in reproduction in term of adult survival [9]. This study represents the first comparative analysis demonstrating this pattern in cyprinid fish species.

Because of the lack of significant relationship between fecundity and k-value, it seems that fish with high growth rate do not invest in high output reproduction (i.e. high fecundity). However, the fecundity used in our comparative study represents an average number of eggs per female in one breeding season, and only such evaluation of fecundity data are available for cyprinids. The measure of fecundity is not an absolute measure of the fish investment in reproductive cells, and it will be useful to obtain the fecundity in relation to average egg size for future comparative studies. K-value from the Von Bertalanffy growth model is considered as a good predictor of mortality [38]. Following Reznick et al. [9] the extrinsic mortality (i.e. mortality attributable to external features of the environment such as disease or predation) is generally considered to be the most important factor shaping the evolution of senescence.

### Fish life traits and parasitism

The impact of parasitism is predicted to increase with the diversity of parasites to which a given host must face, and therefore parasite species richness may be considered as a good predictor for evaluating how the evolution of host life history traits is shaped by parasitism [39-41]. Host natural mortality, estimated by k-value of the Von Bertalanffy growth model, was recognized as a factor contributing to the variability of parasite species richness. Moreover, parasite species benefit from long-lived fish as showed by previous studies (see [39,41] and references therein).

### Parasitism, immunity and reproduction: intra-specific analyses

Trade-off between investment in immune function and cost of parasites during fish spawning has been predominantly investigated following Hamilton and Zuk's [35] hypothesis [6,36,42-44]. In the case of the Arctic charr, *Salvelinus alpinus *(L.) [7], the cost due to parasitism was predominantly confirmed in fish males. Spawning males (i.e. reproductive) were more susceptible to parasite infection than resting males (i.e. non-reproductive) [7]. This finding is in accordance with the immunohandicap hypothesis [29].

In the present study, the relative investment between immune function and reproduction has been investigated mainly on fish males at intra-specific level. Here, we found a significant effect of parasitism on gonad weight in the males of four fish species, but no effect on spleen weight. At the intra-specific level Taskinen and Kortet [44] did not find any relationship between immunocompetence (measured by spleen size) and sexual ornamentation in roach, *Rutilus rutilus*, but found that host resistance against the most prevalent and abundant parasite species (and measured by the proportion of dead parasites) was positively correlated with ornamentation in males. The observed relationship between level of steroid hormones and sexual ornamentation in roach is consistent with the predictions of the immunohandicap hypothesis [37]. However, studies comparing levels of steroid hormones and immune variables are still scarce [13]. Nevertheless, a lack of association between spleen size and either sexual ornamentation (breeding tubercles in common bream) or parasite infection did not seem to support the immunohandicap hypothesis [20]. The relative spleen size in fish is recently used as an organ reflecting an immune investment against parasites or pathogens [7,20]. The negative relationship between spleen size (as a measure of immunocompetence) and condition factor was recorded and interpreted as a trade-off between investment in immune response and somatic condition [20]. This observation suggests that the spleen size is a reliable measure of investment in immune response [20]. Skarstein et al. [7] suggested that a large spleen in fish can be interpreted either as an improving ability to respond to parasite exposure or an indication of high immunological activity from already established infection.

### Parasitism, immunity and reproduction: inter-specific analyses in males

Our results based on inter-specific comparative analyses are not in accordance with the immunohandicap hypothesis [29]. Our field investigation was performed only on reproductive males during the breeding period. If we consider that the expression of secondary sexual traits initiated by the production of testosterone is related to higher investment in gonad size, then high parasite infection should stimulate immune function related with decreased investment in gonad size. A trade-off between investment in reproduction and immune defence should be more evidenced in breeding period with increasing effort in reproduction. However, our results showed a lack of relationship between gonad size and spleen size in fish males, which does not seem to be biased by sampling size as the same results were obtained including the whole fish sample or excluding fish with low sampling size. Moreover, we should note that several comparative analyses using spleen size measurements in birds gave consistent results even if based on low sample size. For instance, half of the bird species in the recent study of Møller et al. [11] are represented by only 1 or 2 individuals.

However, until now no study has provided good evidence that spleen size is related to humoral immune function in fish. Future comparative studies are needed including other immune variables such as plasma IgM concentration, cell-mediated immune response, migratory and phagocytotic activity of head kidney granulocytes representing their functional activity, which have been applied in the recent intra-specific studies of fish immunocompetence [13,19,45].

A relationship between parasitism and sexual dichromatism was observed in fish at the inter-family level, but cyprinid species seem to deviate from this pattern [42]. This should by explained by the fact that many cyprinid species did not express secondary sexual characters, i.e. in our study only a few cyprinid species show measurable sexual traits that could be evaluated at the inter-specific level, and with one exception there is no parental care in the sampled cyprinid species.

### Parasitism, immunity and reproduction: inter-specific analyses in females

We found a negative relationship between spleen weight and gonad weight in females during the breeding period using comparative analyses, supporting the energetic trade-off hypothesis, i.e. a cost in reproduction associated with an investment in immune function. As this result was confirmed from all analyses, i.e. also from the analysis excluding the effect of low sample size and from both phylogenetically corrected or non-corrected analyses, we suggest that this could reflect the evolutionary life-history allocation of energetic resources in cyprinid females. However, the relationship between parasite abundance and spleen weight could suggest that the increasing in spleen weight, i.e. high investment in immune function, represents a response to high exposition of ectoparasites (corresponding to the higher intensity of infection by monogenean parasites of the *Dactylogyrus *and *Gyrodactylus *genera). This suggests that highly parasitized females develop larger spleen as a higher investment in immune defence.

Brown and Brown [10] investigated in birds whether larger spleens reflect the evolution of greater immune investment as life-history strategy or whether represent a proximal response to current exposure to parasites. We observed that parasite abundance or parasite species richness obtained from field data are correlated with data on parasite species richness (Spearman correlation coefficient, p < 0.05) obtained from the regional data analysis of Šimková et al. [46]. It means that parasite abundance could be considered as a reliable measure of parasite pressure, integrating the information on both species richness (diversity) and number of individuals per species. Moreover, such an integrative variable based on a composite sampling, here abundances of several parasite species, is more statistically effective when variance is greater than the mean in each variable, which is the case as fish parasites are over-dispersed (fitting negative binomial distribution). Therefore, our results suggest that spleen weight is not a simple reflection of a current parasite level, i.e. a proximal effect. Moreover, there was a trend of difference in spleen weight corrected for fish weight (KW test, p = 0.07) when comparing females and males with the males having the larger spleen. The similar observation was demonstrated in roach, *Rutilus rutilus *[13].

## Conclusion

We interpret our results in the context of life-history theory, with host species encountering more parasites should invest more extensively in immune defence, i.e. ultimate or evolutionary effect, as it was already found in birds and their ectoparasites [2] or endoparasites [4]. Our study shows for the first time that this kind of relationship could occur in cyprinid female fish, but not in cyprinid fish males. Moreover, we show a parasite impact on female reproductive potential suggesting fitness costs due to parasitism. This effect was previously demonstrated in several studies on birds [47,48] and represents a traditional explanation for negative fitness consequences of parasitism. On the other hand, our prediction related to the immunohandicap hypothesis was not confirmed for fish males.

## Methods

### Data from literature

Data on 23 cyprinid fish species from Central Europe were used. Data on the following life-history traits were obtained: maximal fish size, female fecundity and maximal longevity [46,49,50]. In those study the estimation of the values of different life history traits are based on published records across Central Europe. Values of k, one parameter of the von Bertalanffy growth function, were obtained from FishBase [38] (Table 1). The k value is related to longevity and is considered as a good predictor of natural mortality [38].

Data on species richness of ectoparasites (including Monogenea, Mollusca, Hirudinea, Copepoda) and endoparasites (including Digenea, Cestoda, Acanthocephala and Nematoda) were obtained from Šimková et al. [46] (Table 1).

### Data from fish sampling

Among 23 fish species, a total of 21 fish species were investigated in a field study conducted in the two last weeks of May 2002, from the Morava river basin (Czech Republic). We limited the sampling period in order to eliminate the confounding effect of water temperature changes. The collection of fish species was also attempted to include the fish in breeding period or near their breeding period. Therefore, our sampling was time-limited in order to collect fish in the period with no fluctuations in water temperature, as the changes in water temperature could affect spleen size, gonad size and the composition of parasite communities [19]. All fish were collected by electrofishing. Fish individuals were separated by sex. The mean total body weight, gonad weight and spleen weight (with their standard deviations) were measured for each individual (data are given in Table 4). Non-breeding individuals were excluded from the analyses and only the individuals at the same gonad maturation stage were retained for analyses. Thus, information was restricted to 49 females of 17 fish species and 79 males of 20 species.

The complete dissection of fish was performed using the method of Ergens and Lom [51]. Fish were examined for all metazoan parasites. Therefore, external organs (fish skin, fins, gills, eyes) and internal organs (intestine, hepatopancreas, spleen, protonephros, heart, swim bladder) were examined for the following groups: ectoparasites (Monogenea, Crustacea, Mollusca and Hirudinea) and endoparasites (Digenea, Nematoda, Cestoda and Acanthocephala). All parasites were counted under dissecting microscope.

We used two measures of parasite impact:

- average parasite species richness, i.e. the average species richness per individual host for a given species, which could be split in endo- and ectoparasites. This measure represents the parasite diversity a given fish species;

- mean total parasite abundance, which is the mean of the sum of all parasite individuals of all parasite species in a given fish species. This composite variable is statistically appropriate when variance is greater than the mean for each variable, which are the cases as fish parasite distribution follows the negative binomial distribution. Total parasite abundance represents an overall measure of parasite pressure.

Those data are given in Table 4.

Data obtained from published resources were not adequate for analysing the potential trade-off between gonad size (measure of reproductive investment) and spleen size (measure of immune function) as they do not provide information in relation to fish sex. Therefore, we used data on parasites directly obtained from the field study separating females and males.

### Intra-specific analyses

We conducted intra-specific analyses on the males of four fish species, for which enough individuals were collected: *Barbus barbus, Leuciscus cephalus, Pseudorasbora parva *and *Rutilus rutilus*.

First, we conducted a composite variance analysis on the males in order to estimate the percentages of variance in spleen weight and gonad weight due to fish species.

Second, we performed ANOVA on GLM to test the influence of average parasite species richness, mean total parasite abundance, body weight on (1) gonad weight and (2) spleen weight. The variables were log transformed prior analyses.

### Comparative analyses

The phylogenetic independent contrasts method was used [52] with the CAIC program for Macintosh [53]. The phylogeny of cyprinid fish species was obtained from the molecular analyses of the combined sequences of cytochrome *b*, 16S and control region of mtDNA [54]. All continuous variables were log-transformed before analysis to achieve homogeneity of variance [55]. We controlled for the non-violation of assumptions of the independent contrasts method [53,56].

We tested (1) the respective importance of fish life history traits in determining total parasite species richness, ectoparasite species richness and endoparasite species richness, (2) the influence of parasite abundance and species richness on spleen and gonad weights by performing a stepwise regression forced through the origin [57] on all independent variables.

Because of the allometric relationship with maximal fish size; female fecundity and maximal longevity were corrected for maximal fish size using residuals of log-transformed life trait versus log-transformed maximal fish size.

## Authors' contributions

AS and SM performed the statistical and comparative analyses, drafted the manuscript and discussed the results. PJ collected the fish specimens. TL, MO and EO participated on fish dissections, collection and determination of parasites. TL also participated on the data treatment and involved in drafting of manuscript. All authors read and approved the final manuscript.
